# CryoAlign2: efficient global and local Cryo-EM map retrieval based on parallel-accelerated local spatial structural features

**DOI:** 10.1093/bioinformatics/btaf296

**Published:** 2025-05-10

**Authors:** Zhe Liu, Bintao He, Tian Zhang, Chenjie Feng, Fa Zhang, Zhongjun Yang, Renmin Han

**Affiliations:** Research Center for Mathematics and Interdisciplinary Sciences, Cheeloo College of Medicine, Qilu Hospital (Qingdao), Shandong University, Qingdao 266237, China; College of Medical Information and Engineering, Ningxia Medical University, Yinchuan 750004, China; Research Center for Mathematics and Interdisciplinary Sciences, Cheeloo College of Medicine, Qilu Hospital (Qingdao), Shandong University, Qingdao 266237, China; Research Center for Mathematics and Interdisciplinary Sciences, Cheeloo College of Medicine, Qilu Hospital (Qingdao), Shandong University, Qingdao 266237, China; College of Medical Information and Engineering, Ningxia Medical University, Yinchuan 750004, China; College of Medical Information and Engineering, Ningxia Medical University, Yinchuan 750004, China; School of Medical Technology, Beijing Institute of Technology, Beijing 100081, China; Research Center for Mathematics and Interdisciplinary Sciences, Cheeloo College of Medicine, Qilu Hospital (Qingdao), Shandong University, Qingdao 266237, China; Research Center for Mathematics and Interdisciplinary Sciences, Cheeloo College of Medicine, Qilu Hospital (Qingdao), Shandong University, Qingdao 266237, China

## Abstract

**Motivation:**

With the rapid advancements in Cryo-Electron Microscopy (Cryo-EM), an increasing number of high-resolution 3D density maps are being made publicly available, highlighting the urgent need for efficient structure similarity retrieval. Exploring map similarity at various levels is critical for fully utilizing these valuable resources. Our previously proposed CryoAlign can provide more accurate density map alignment while maintaining a low failure rate. However, CryoAlign only offers a method for aligning density maps, with low efficiency in local alignment, and has not yet been applied to the retrieval of Cryo-EM density maps.

**Results:**

We have developed an alignment-based retrieval tool to perform both global and local retrieval. Our approach adopts parallel-accelerated CryoAlign for high-precision 3D alignment and transforms density maps into point clouds for efficient retrieval and storage. Additionally, a multi-dimension scoring function is introduced to accurately assess structural similarities between superimposed density maps. To demonstrate its applicability, we conducted thorough testing across different retrieval tasks, such as global, local or hybrid similarity retrieval. Our tool achieves up to a 7-fold speedup while supporting precise local alignments. Comprehensive experiments demonstrate that even when one density map is entirely contained within another, our tool performs exceptionally well in high-resolution density map retrieval. It provides researchers with an efficient and accurate solution for density map similarity search.

**Availability and implementation:**

The source code, documentation, and sample data can be downloaded at https://github.com/JokerL2/CryoAlign2.

## 1 Introduction

The recent developments in Cryo-electron microscopy (Cryo-EM) technology are advancing the determination of 3D structures of complex macromolecules (Chen Bai *et al.* 2015). An increasing number of high-resolution biological macromolecular structures have already been resolved and stored in public databases such as the Electron Microscopy Data Bank (EMDB) (Lawson *et al.* 2016). As of December 2024, EMDB contains over 40 000 density map entries, with maps at resolutions ranging from 2 to 8 Å accounting for 78.9% of the total. Searching maps of similar structures in the database is the most fundamental application of these valuable resources. Effective structure-based retrieval aids researchers in collecting protein with potential similar functions and exploring structural changes in different environments (Herreros *et al.* 2023). For complex macromolecules with composite structures, global shape similarity is not sufficient for the demands of function retrieval, in which local structural similarity dominates (Frank 2021, Si *et al.* 2023). However, the local regions of medium and high-resolution density maps are relatively complex, making it challenging to direct capture corresponding structural features between two unaligned maps. Especially the query map can be fully embedded within the candidate map (Zhong *et al.* 2021).

Currently, a couple of methods have been developed for EM map database retrieval. One of them is Omokage (Suzuki *et al.* 2016), which models maps with Gaussian mixture model (GMM) and obtains the correlation of two GMM functions using the gmfit program. EM-SURFER (Han *et al.* 2017), a web-based tool for real-time global EM map database retrieval, adopts 3D Zernike descriptors (3DZD) (Novotni and Klein 2003, Kihara *et al.* 2011, Pintilie and Chiu 2021) to capture the similarity between the map isosurfaces. Both tools provide extremely fast global retrieval, capable of searching the entire EMDB in minutes or seconds. However, the global shape features extracted by these methods primarily emphasize the overall characteristics of the map, making them inadequate for identifying partial similarities. This limitation in detecting local structural features restricts their applicability in more complex or region-specific retrieval tasks (Kawabata 2008).

Alignment-based approaches evaluate similarities between superimposed maps to eliminate interference caused by significant differences in molecular weight. Fitmap is a map fitting module in ChimeraX (Pettersen *et al.* 2004), a widely used software for molecular manipulation, and provides correlation calculation between two aligned maps. Although the combination of accurate fitting and correlation is sufficient for retrieval tasks, it requires reliable initial positions provided by users, making it unsuitable for fully automated workflows. Han *et al.* (2021) propose a vector-based architecture called VESPER for global and local alignment and introduces a specialized *Z*-score to calculate map similarity. However, VESPER relies on an exhaustive retrieval to determine rotation and translation parameters, which significantly limiting both the accuracy and speed of alignment. For density maps larger than 500 MB, this method takes more than 5 minutes on a single core, making it less suitable for complex retrieval tasks. In contrast, CryoAlign (He *et al.* 2024) models density maps as 3D point clouds, enabling fast and high-precision alignment while maintaining a lower failure rate. More accurate alignments provide a stronger foundation for map searching. Unfortunately, CryoAlign is solely an alignment method without a built-in similarity judgment, and its corresponding Python-implementation has relatively low computational resource utilization.

Here, we proposed an alignment-based Cryo-EM density map retrieval tool that models 3D volumes as point clouds and uses parallel-accelerated CryoAlign2 for accurate global and local alignment. A scoring function is further built based on the superimposed maps, integrating multiple levels of structure similarity including geometric error, feature correlation, and point cloud distribution. This comprehensive similarity score supports global, local and hybrid retrieval patterns and is used to identify the top-K retrieval results. In addition to the retrieved maps, our tool also provides corresponding alignment parameters, assisting users in locating regions with partial similarity. Compared to existing retrieval tools, our approach demonstrates superior performance in handling complex retrieval scenarios.

## 2 Materials and methods

### 2.1 Overview of the retrieval architecture

**Figure 1.**
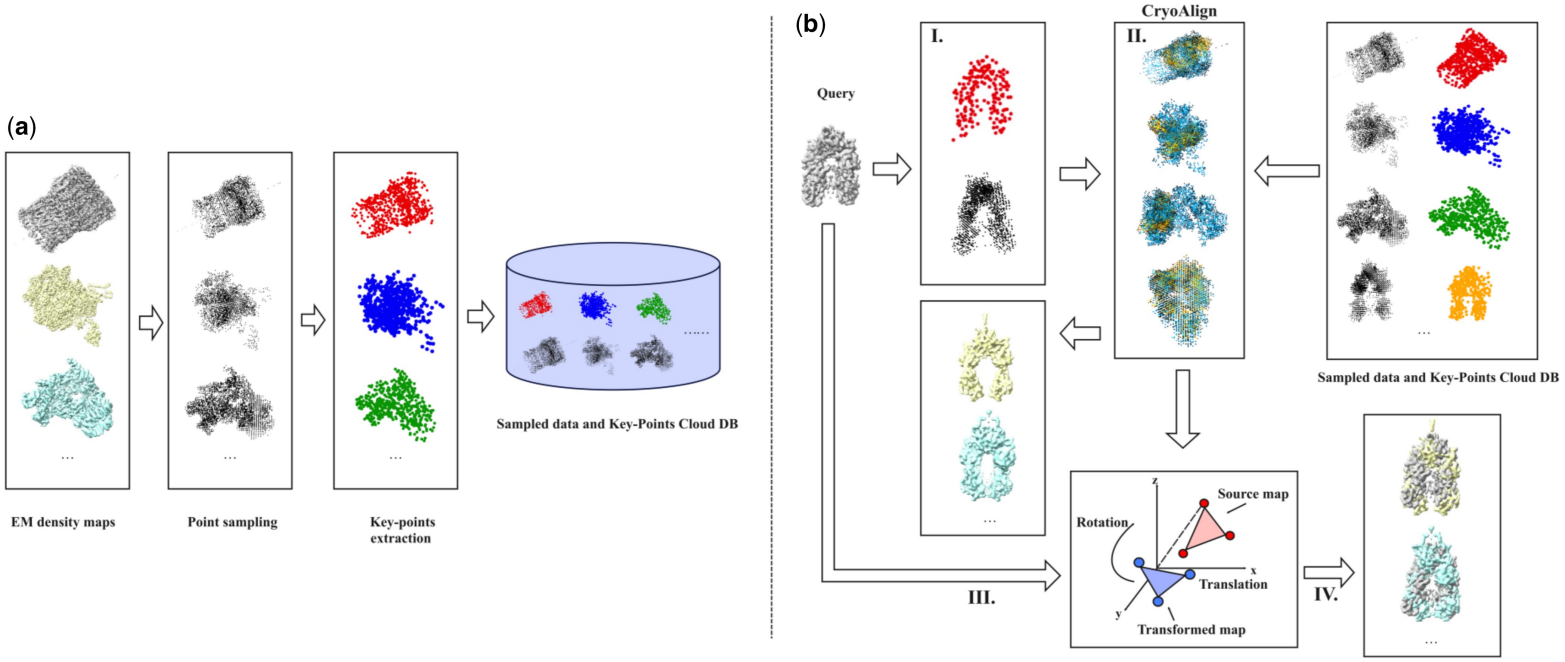
Overview of the CryoAlign retrieval procedure. (a) Automated construction of the key point cloud library for EM maps. This process includes batch input of EM density map MRC files, sampling the density maps to extract key points from the sampled point clouds, and storing both key points and sampled points in the library. (b) CryoAlign-based retrieval procedure for Cryo-Electron Microscopy Density Maps, comprising four main steps: (I) Input and processing of the query map, including sampling and key point extraction. (II) Matching the query map against all point clouds in the key point cloud library using CryoAlign, evaluating registration results with a scoring function, and ranking the results to identify the top 10 best rotation-translation (RT) transformation matrices and corresponding maps. (III) Application of the top 10 RT transformation matrices to the query map to generate the transformed query maps. (IV) Visualization of the transformed query maps alongside the top 10 similarity score maps using ChimeraX for overlay comparison.

CryoAlign-based retrieval application facilitates efficient density map retrieval by constructing a robust retrieval architecture. Figure 1 illustrates the workflow of our retrieval application, including the construction of the retrieval database and the retrieval process. The process begins with the creation of a database for CryoAlign-based matching, as illustrated in Fig. 1a. This involves sourcing raw Cryo-EM density maps from EMDB or customized datasets. For each input density map, the process involves multiple steps, including sampling and clustering. Specifically, the map is first converted into vectors, and from the voxel values and these vectors, the 3D point cloud data of the entire density map are obtained. Subsequently, these point cloud data are clustered using the Meanshift (Carreira-Perpinan 2006) and DBSCAN (Ester *et al.* 1996) algorithms to extract the key points of the density map. These key points, extracted from all density maps, and point cloud data are then saved collectively to form the retrieval database. Notably, this approach leverages the principle that CryoAlign converts the challenging task of density map matching into a 3D point cloud registration problem. In the retrieval application, the target map is matched against all maps in the database. By pre-saving the key points, the computational process during retrieval is streamlined since only the extraction of key points from the target map is required. This design significantly boosts retrieval efficiency. Furthermore, because the final stored format consists of point cloud data derived from the density maps, the database remains compact, which enhances its portability and simplifies its maintenance.

The core CryoAlign workflow for similarity comparisons between pairs of Cryo-EM density maps is composed of several critical steps. First, the initial point cloud is sampled at a specified interval, and density vectors for all points are calculated. Following this, a clustering algorithm is applied to extract key points that represent the coarse structural skeleton of the density map. These key points play a pivotal role as they enable the computation of local spatial structure descriptors, which effectively capture the local features surrounding the key points. CryoAlign then utilizes these extracted descriptors and applies mutual feature matching techniques to efficiently and robustly estimate the initial pose parameters. Finally, the optimal alignment between density maps is achieved through an iterative process that minimizes the distance between corresponding points. One of CryoAlign’s significant advantages lies in its ability to decouple the key point extraction process from the point cloud registration process. This modularity is particularly beneficial as it permits the precomputation of key point extraction, enabling the persistent storage of point cloud data. This design choice markedly enhances the efficiency of density map similarity retrieval. Conversely, if these processes were tightly coupled, the efficiency of retrieval would be severely hindered. By generating feature descriptors for the density map through key point extraction, CryoAlign ensures that retrieval applications can be performed efficiently and at scale. As a result, the retrieval architecture benefits greatly from this foundational step, allowing for rapid and accurate similarity comparisons.

The retrieval process is further detailed in Fig. 1b, which outlines the workflow for Cryo-EM density map retrieval. When a query map is entered, it is first transformed into point cloud data. This point cloud is then matched against each point cloud in the database using CryoAlign. The outcomes of each point cloud registration are evaluated based on a set of criteria, including geometric error, cosine similarity of normals, consistency of point cloud density, and similarity of SHOT features. These individual criteria are combined into a normalized composite score that indirectly reflects the similarity between the Cryo-EM density maps. Lastly, all comparison results are ranked, and the top 10 density maps are identified based on their scores. Additionally, RT matrices generated from the pairwise point cloud data comparisons are provided, which can be used for target-source overlay visualizations.

Details of all methods and experiments can be found in the Supplementary Materials, available as supplementary data at *Bioinformatics* online, which include Section 1: METHOD, available as supplementary data at *Bioinformatics* online and Section 2: EXPERIMENTS, available as supplementary data at *Bioinformatics* online.

### 2.2 Similarity scoring function

Our framework assesses Cryo-EM density map alignment quality through a multi-metric scoring function based on the rotation-translation (RT) matrix derived from point cloud registration. The scoring system combines: Geometric displacement error, Surface normal cosine similarity (Wang *et al.* 2023), Volumetric density consistency (Chen *et al.* 2023), and Signature of Histograms of Orientations (SHOT) similarity feature (Salti *et al.* 2014). The pipeline comprises two principal stages: First, CryoAlign computes the optimal RT matrix by rigidly transforming the source point cloud to target coordinates. Second, quantitative measures are computed across these four dimensions to evaluate alignment fidelity from complementary perspectives (detailed in Section 1.A, available as supplementary data at *Bioinformatics* online).

### 2.3 Parallel strategy for alignment

When constructing a Cryo-EM density map database, CryoAlign aligns target and source maps by matching key point clouds. To avoid wasting time resampling point clouds for each alignment, density maps are precomputed and stored in the database. During retrieval, only the target map is sampled, which significantly reduces retrieval time. A hybrid parallel strategy is used for database construction: (i) To enhance the efficiency of point sampling and map comparison, memory mapping is used for bit-by-bit reading of dimensions and voxel values, combined with multiprocessing to achieve coarse-grained optimization. (ii) The primary performance bottlenecks in the point sampling process reside in the feature extraction phase of the Meanshift algorithm and the GPU-CPU data transfer. Our analysis revealed that while GPU-based convolution operations improve computational efficiency, the gains are offset by the overhead of GPU-CPU data transmission. To address this, we strategically relocated feature extraction to the CPU, thereby eliminating GPU-CPU transfers and implementing MPI-based parallel acceleration. Concurrently, we applied fine-grained parallelization to optimize the iterative computations of the Meanshift algorithm (see Section 1.B and Algorithm 1, available as supplementary data at *Bioinformatics* online for implementation details). (iii) The local point cloud registration process, which inherently involving an iterative traversal mechanism, is further optimized through fine-grained parallelization of the registration algorithm, ensuring improved computational efficiency and scalability(see more details in Section 1.B and Algorithm 2, available as supplementary data at *Bioinformatics* online).

## 3 Results

To assess the performance of our retrieval system, we collected low-temperature electron microscopy images from a dataset provided by VESPER, specifically curated for both global and local density map retrieval (Joseph *et al.* 2017). Initially, we excluded images with a resolution below 10 Å, focusing instead on those with a resolution above 10 Å and ensuring that each class contained more than five density maps. This selective filtering resulted in two distinct datasets for evaluating retrieval performance: the global matching retrieval dataset, which includes 75 Cryo-EM density maps across 15 classes, and the local matching retrieval dataset, featuring 181 Cryo-EM density maps across 24 classes. In addition, we achieved cross mixing of tasks between global and local matching retrieval to comprehensively evaluate the effectiveness and robustness of retrieval applications (see more details in Section 2.A and Table 1, available as supplementary data at *Bioinformatics* online).

**Table 1.** Performance comparison under different global/local retrieval ratios.

Global/local ratio	Alignment methods type	Precision	Recall	F1 score	Run time (6000 times)
20%/80%	EM-SURFER(3D Zernike)	66.37%	46.44%	54.66	**4 min**
	Omokage(gmfit)	78.25%	50.58%	61.58	17 min
	VESPER	**99.46**%	75.88%	86.09	1 h 34 min
	CryoAlign2(Local)	99.36%	84.47%	91.32	32 min
	CryoAlign2(Global)	99.43%	80.43%	88.92	22 min
	CryoAlign2(Hybrid)	99.39%	**88.31%**	**93.52**	45 min
50%/50%	EM-SURFER(3D Zernike)	74.58%	67.26%	70.44	**3 min**
	Omokage(gmfit)	82.38%	77.61%	80.06	12 min
	VESPER	98.26%	76.53%	86.00	1 h 22 min
	CryoAlign2(Local)	99.21%	85.49%	91.92	30 min
	CryoAlign2(Global)	99.33%	81.58%	89.70	18 min
	CryoAlign2(Hybrid)	**99.47%**	**88.57%**	**93.70**	37 min
80%/20%	EM-SURFER(3D Zernike)	81.71%	76.32%	78.86	**5 min**
	Omokage(gmfit)	85.64%	77.88%	81.69	21 min
	VESPER	99.31%	82.14%	89.90	1 h 35 min
	CryoAlign2(Local)	99.36%	84.47%	91.25	35 min
	CryoAlign2(Global)	99.29%	83.17%	90.52	24 min
	CryoAlign2(Hybrid)	**99.46%**	**89.11%**	**93.66**	46 min

The bold data is the best result of the test indicators.

Table 1 demonstrates retrieval performance comparisons under different data distributions. To test CryoAlign2’s capability in handling hybrid global-local retrieval tasks, the dataset was constructed by proportionally mixing global and local retrieval tasks, comprising 15 classes with 5 mutually similar density maps per class. Approximately 6000 pairwise comparisons were conducted, where each density map was compared against all maps within its own class and across other classes. The first column specifies the global/local ratio and alignment method type. CryoAlign2 provides three alignment strategies. CryoAlign2 (Global): exclusively performs global alignment during map comparisons. Global alignment delivers the fastest execution speed by performing rapid similarity comparison between two density maps; CryoAlign2 (Local): executes only local alignment, Local alignment operates at a comparatively slower speed (detailed in Section 1.B, available as supplementary data at *Bioinformatics* online); CryoAlign2 [Hybrid (Global and Local)]: combines both global and local alignment methods by averaging their scores, Hybrid alignment, while requiring the longest processing time, achieves enhanced precision through integration of global and local features. The first column of each table lists the Alignment Methods Type, followed by columns showing precision, recall, F1-score, and the time required to retrieve 75 density maps after 6000 comparisons. Our experimental approach uses a cross-dataset strategy that encompasses both global and local retrieval tasks. The first line reveals that global retrieval tasks account for 20% of the density maps, while local retrieval tasks constitute 80%. With a predominance of local retrieval task data in the database, EM-SURFER and Omokage exhibit a significant number of errors, resulting in low F1 scores. Although EM-SURFER demonstrates the fastest matching speed, the inclusion of extensive local retrieval data significantly compromises its precision. The second line outlines a scenario where global and local retrieval density maps each comprise 50% of the dataset. As the proportion of global retrieval maps increases and that of local retrieval maps decreases, the retrieval accuracy for both EM-SURFER and Omokage improves. However, their performance is still adversely affected by the presence of local retrieval maps. The third line shows that when the dataset consists of 80% global retrieval maps and only 20% local retrieval maps, the retrieval accuracy for EM-SURFER and Omokage significantly increases compared to predominantly local retrieval tasks. Nevertheless, their performance is still influenced by the proportion of local maps. This underscores that EM-SURFER and Omokage are less effective for tasks involving a high proportion of local retrievals, and their accuracy diminishes in hybrid retrieval scenarios.

In contrast, our tool, which uses the Global method, demonstrates a speed that matches Omokage, yet the inclusion of local retrieval task data minimally affects the overall retrieval accuracy. This confirms that our application is capable of efficiently handling both hybrid retrieval tasks. Our application achieves its highest F1 score when using a combination of Hybrid(Global and Local) method. However, the Local method, which involves traversing the entire point cloud based on the movement step size, is significantly slower than Global method, even when accelerated by parallel computing. Despite this, its retrieval speed remains quicker than that of the VESPER matching method, which requires nearly 1.5 h to perform the same 6000 comparisons.

To evaluate the performance of our retrieval application, we conducted a series of experiments, including assessing the effectiveness of the scoring function (Section 2.B, Figs 2 and 3, available as supplementary data at *Bioinformatics* online), the impact of alignment methods and scoring functions (Section 2.c, Table 3, available as supplementary data at *Bioinformatics* online), the retrieval of unknown category density maps (Section 2.D, Fig. 4, available as supplementary data at *Bioinformatics* online), the correct alignment promotes accurate search (Section 2.E: Fig. 5, available as supplementary data at *Bioinformatics* online), the robustness of our retrieval tool (Section 2.F: Fig. 6, available as supplementary data at *Bioinformatics* online) and an analysis of running time (Section 2.G: Figs 7 and 8 and Table 4, available as supplementary data at *Bioinformatics* online). The experimental results indicate that our retrieval application outperforms existing approaches by achieving a superior balance of efficiency and accuracy.

## 4 Conclusion

In this work, we developed a robust and high-performance Cryo-EM density map retrieval application based on CryoAlign, encompassing the automated construction of retrieval libraries, optimization of CryoAlign’s local alignment methods, and the design of a novel Multi-score evaluation metric. The multi-dimension score metric enables comprehensive assessment of alignment results from multiple perspectives, enhancing the reliability and accuracy of both global and local retrieval tasks. Our application effectively distinguishes between density maps of the same macromolecular class and those with structural similarity and offers three distinct retrieval methods tailored for varying precision and efficiency requirements. For high-accuracy results, a combination of Hybrid(Global and Local) method is recommended, while for high-speed retrieval, Global method suffices.

Significant improvements in computational efficiency were achieved through parallel optimization, with local alignment achieving a 7x speedup compared to single-thread performance. However, resource contention may occur with excessive threading, and retrieval performance for large-scale datasets remains constrained by inherent computational demands. Hence, we recommend leveraging the application for smaller datasets or targeted retrieval tasks to maximize efficiency.

To address challenges posed by volume discrepancies and resolution variations, we enhanced the scoring function from a single normal cosine similarity approach to a multi-dimension scoring design. This improvement significantly bolstered the robustness of the retrieval process, particularly in local search tasks, which are inherently more challenging due to substantial volume differences between source and target maps. The multi-dimension evaluation successfully mitigates issues such as inflated similarity scores caused by map enveloping, ensuring more accurate alignment even for maps with high-resolution, feature-rich local structures or low-resolution, sparsely sampled features.

Overall, our retrieval application proves to be a valuable tool for efficiently constructing point cloud retrieval libraries and performing fast, accurate similarity-based searches for Cryo-EM density maps. It supports researchers in analyzing complex macromolecular structures by providing precise alignment results and leveraging RT transformation matrices for subsequent map fitting.

## Supplementary Material

btaf296_Supplementary_Data
