# Supplementary material for: CryoAlign2: efficient global and local Cryo-EM map retrieval based on parallel-accelerated local spatial structural features
Source: Bioinformatics. 2025 May 10;41(5):btaf296. doi: 10.1093/bioinformatics/btaf296 (PMC12122211; doi:10.1093/bioinformatics/btaf296)
Supplement: btaf296_Supplementary_Data [file btaf296_supplementary_data.zip › Supplementary Materials for Online.pdf]

1. METHODS

A. Similarity Scoring Function

Our framework evaluates the alignment quality of Cryo-EM density maps through a comprehensive scoring function based on the RT matrix, which encapsulates rotation and translation, obtained from point cloud registration. The scoring function integrates multiple metrics, including geometric error, normal cosine similarity Wang et al. (2023), point cloud density consistency Chen et al. (2023), and SHOT feature similarity Salti et al. (2014), to provide a robust and multi-faceted evaluation. Initially, CryoAlign computes the optimal RT matrix that rigidly transforms the source point cloud to align with the target. Subsequently, various metrics are calculated to assess the similarity from different perspectives.

The geometric error (d_{geo}) quantifies the spatial alignment between the source and target point clouds. It is defined as the mean Euclidean distance between the transformed points in the source point cloud $\mathbf{a}'_i$ and their corresponding points in the target point cloud $\mathbf{b}_j$, where K is the total number of point correspondences. This metric captures the positional deviation between the two point clouds.

$$d_{\text{geo}} = \frac{1}{K} \sum_{i=1}^{K} \|\mathbf{a}'_i - \mathbf{b}_j\|_2 \tag{S1}$$

Normal similarity ($\text{NS}(A, B)$) evaluates the alignment of surface orientations between the source and target point clouds. It is determined by computing the cosine similarity of the normal vectors $\mathbf{n}_{\mathbf{a}_i}$ and $\mathbf{n}_{\mathbf{b}_j}$, weighted by a correspondence weight w_i. Normal similarity reflects the directional consistency of local surface features.

$$\text{NS}(A, B) = \frac{\sum_{i=1}^{K} \left(\frac{\mathbf{n}_{\mathbf{a}_i} \cdot \mathbf{n}_{\mathbf{b}_j}}{\|\mathbf{n}_{\mathbf{a}_i}\| \|\mathbf{n}_{\mathbf{b}_j}\|} \cdot w_i \right)}{\sum_{i=1}^{K} w_i} \tag{S2}$$

Density consistency ($\text{DC}(A, B)$) assesses the alignment of local densities between the source and target point clouds. It evaluates the consistency of neighborhood structures by comparing the number of neighboring points within a predefined radius r, thereby providing insights into differences in local density distributions. This metric is particularly valuable for identifying misalignments due to sampling variations, noise, or resolution inconsistencies. Here, $\text{Nbr}_A(p_i, r)$ and $\text{Nbr}_B(q_i, r)$ represent the number of neighboring points within radius r for points p_i and q_i in the source and target point clouds, respectively.

$$\text{DC}(A, B) = \frac{1}{K} \sum_{i=1}^{K} |\text{Nbr}_A(p_i, r) - \text{Nbr}_B(q_i, r)| \tag{S3}$$

Signature of Histograms of Orientations (SHOT) feature similarity ($\text{SHOT}(A, B)$) evaluates the local geometric similarity between the source and target point clouds by computing the cosine similarity of SHOT descriptors $\mathbf{f}_{\mathbf{a}_i}$ and $\mathbf{f}_{\mathbf{b}_j}$ across all correspondences, where K denotes the total number of correspondences.

$$\text{SHOT}(A, B) = \frac{1}{K} \sum_{i=1}^{K} \frac{\mathbf{f}_{\mathbf{a}_i} \cdot \mathbf{f}_{\mathbf{b}_j}}{\|\mathbf{f}_{\mathbf{a}_i}\| \|\mathbf{f}_{\mathbf{b}_j}\|} \tag{S4}$$

Each metric is normalized to ensure its value lies within the interval $[0, 1]$, thereby facilitating fair integration into the final score. The final similarity score is computed as the weighted sum of the normalized scores, w reflecting the relative importance of each metric:

$$\text{final\_score} = w_1 \cdot \text{nor}_{\text{NS}} + w_2 \cdot \text{nor}_{\text{SHOT}} - w_3 \cdot \text{nor}_{\text{d\_geo}} - w_4 \cdot \text{nor}_{\text{DC}} \tag{S5}$$

w_1 is used to assess the consistency of surface normals. Since normal information reflects the local geometric structure of the point cloud, this metric is given a high weight to enhance sensitivity to local alignment characteristics. w_2 indicates the degree of matching in local geometric features. In the case of high-resolution Cryo-EM point clouds, the registration of local features is particularly important, which is why this weight is also assigned a high value. Furthermore, w_3 evaluates the global alignment of point clouds in space. This metric is vital for the overall quality of registration, although it may be influenced by noise or unevenly distributed points in

the cloud, leading to a moderate weight assignment. Lastly, w_4 reflects the differences in local density within the point cloud. Given that density distribution can be affected by data sampling or noise, this metric is assigned a lower weight to reduce unnecessary interference.

The proposed scoring function provides a robust and holistic evaluation of the alignment quality between Cryo-EM density maps. By integrating multiple metrics, such as geometric alignment, consistency of surface orientation, local density properties, and descriptor-based similarity, this framework effectively captures both global and local structural features of point clouds. This comprehensive assessment ensures accurate and reliable retrieval of Cryo-EM density maps across various search scenarios, thereby supporting applications in structural biology and molecular modeling.

B. Parallel Strategy for Extracted Points

Our parallel MeanShift algorithmS1 addresses the critical bottleneck in conventional GPU implementations: the prohibitive latency of PCIe data transfer between GPU and CPU during convolutional operations. Through empirical analysis, we observed that for large-scale density maps like grid sizes of 512*512*512, the data transfer time required to move 3D convolution results from GPU to CPU exceeds the direct CPU-based convolution time by a factor of 2-3. To resolve this, we propose a distributed memory parallelization scheme using Message Passing Interface (MPI) that optimizes data locality while preserving algorithmic accuracy.

B.1. Parallel MeanShift Formulation

The parallel MeanShift algorithm operates on a 3D voxel grid with dimensions $D \times H \times W$ and physical spacing $(\Delta_x, \Delta_y, \Delta_z)$. At its core lies the computation of two fundamental tensor fields that drive the density-aware shifting process. The first tensor $Q \in \mathbb{R}^{D\times H\times W}$ encodes normalized electron density values, where each element $Q[i,j,k]$ quantifies the relative density at voxel (i,j,k). The second tensor $X \in \mathbb{R}^{D\times H\times W\times 3}$ explicitly stores the physical coordinates of voxel centers, defined as $X[i,j,k] = (i\Delta_x, j\Delta_y, k\Delta_z)^T$.

The algorithm's numerical foundation emerges from two Gaussian-weighted convolution operations that generate the numerator $\mathcal{N}$ and denominator $\mathcal{D}$. The numerator $\mathcal{N} \in \mathbb{R}^{D\times H\times W\times 3}$ captures the weighted spatial distribution of density-mass products, computed through the vector convolution $\mathcal{N} = (Q \odot X) * W$, where $\odot$ denotes element-wise multiplication and $*$ the 3D convolution operator. The denominator $\mathcal{D} \in \mathbb{R}^{D\times H\times W}$ represents the density-weighted kernel responses, calculated as $\mathcal{D} = Q * W + \epsilon$, with $\epsilon = 10^{-6}$ ensuring numerical stability. The Gaussian kernel $W \in \mathbb{R}^{w\times w\times w}$ implements spatial weighting with bandwidth σ, formulated as $W[i,j,k] = \exp(-1.5\|(i,j,k) - c\|^2/\sigma^2)$ where c marks the kernel center.

These intermediate results combine through element-wise division to produce the shift vector field $C = \mathcal{N} \oslash \mathcal{D}$, where $\oslash$ performs per-voxel vector normalization. Each $C[i,j,k] \in \mathbb{R}^3$ encodes the optimal shift direction for voxel (i,j,k), weighted by local density gradients. This formulation enables parallel computation by decoupling the spatially invariant convolution operations from the point-wise shift applications.

B.2. Distributed Computation Framework

The implementation employs a master-worker architecture that separates global convolution computations from distributed point processing. The master process (Rank 0) initializes the computation by constructing the Gaussian kernel using physical spacing parameters $\Delta_x, \Delta_y, \Delta_z$, then calculates $\mathcal{N}$ and $\mathcal{D}$ through FFT-accelerated 3D convolution. These tensors remain memory-resident on the master to serve subsequent shift vector queries, while the derived shift field C broadcasts to all workers through MPI_Bcast to maintain consistency.

Point cloud processing follows a cyclic decomposition strategy where the initial point set $Y^{(0)}$ partitions into K chunks (K being the number of MPI processes). Each worker process k receives a contiguous block $Y_k = \{y_i | \lfloor iN/K \rfloor = k\}$ where N is the total point count. During iteration t, workers compute local shift vectors by querying the cached C field through nearest-grid indexing:

$$\Delta Y_k^{(t)}[i] = y_i^{(t)} - C\left[\left\lfloor \frac{y_i^{(t)}}{\Delta} \right\rceil\right]$$

where $\lfloor \cdot \rceil$ implements rounding to nearest voxel coordinates. A magnitude threshold $\theta = lower\_bound^2$ stabilizes the updates by nullifying shifts below the noise floor:

$$\Delta Y_k^{(t)}[i] = \begin{cases} 0 & \text{if } \|\Delta Y_k^{(t)}[i]\|^2 < \theta \\ \Delta Y_k^{(t)}[i] & \text{otherwise} \end{cases}$$

B.3. Communication-Aware Reduction Protocol

The parallel update mechanism employs a three-stage reduction process to minimize synchronization overhead. First, intra-node reductions aggregate partial sums from multiple CPU threads using cache-aligned buffers and Kahan compensated summation. Second, inter-node reductions utilize MPI_Allreduce with a binary tree topology, automatically selecting between FP16 and FP32 precision based on vector magnitudes - vectors with $\|\Delta Y\|_\infty < 10^3$ compress to FP16 format using stochastic rounding, while others retain full FP32 precision.

The global update rule combines these reduced quantities through adaptive normalization:

$$Y^{(t+1)} = Y^{(t)} - \eta \frac{\Delta Y^{(t)}}{\|\Delta Y^{(t)}\| + \epsilon} \odot \min(\|\Delta Y^{(t)}\|, \delta_{max})$$

where η denotes the learning rate and δ_{max} the maximum permissible displacement per iteration. The denominator term $\|\Delta Y^{(t)}\| + \epsilon$ prevents division by zero while maintaining directionality of small shifts.

Algorithm S1. Parallel MeanShift Algorithm with MPI

Master Process:
Load voxel data and compute intervals $\{interval_x, interval_y, interval_z\}$
Create grid tensor $X \in \mathbb{R}^{D \times H \times W \times 3}$
Construct density tensor $Q \in \mathbb{R}^{D \times H \times W}$
Compute background field: $P = Q \odot X$
Generate Gaussian kernel W with $window^3$ size
Compute numerator: $\mathcal{N} = \text{Conv3D}(P, W)$
Compute denominator: $\mathcal{D} = \text{Conv3D}(Q, W) + \epsilon$
Calculate shift vector: $C = \mathcal{N} \oslash \mathcal{D}$
Broadcast C, grid parameters and initial points Y to all workers
All Processes:
Partition point cloud Y into chunks $\{Y_k\}_{k=1}^K$ using block distribution
for each iteration $t \in [1, T]$ **do**
    Initialize $local\_diff \leftarrow 0$, $local\_sq\_sum \leftarrow 0$
    for each point $p_i \in Y_k$ **do**
        Compute grid indices: $idx_x, idx_y, idx_z = \lfloor (p_i - \mu)/interval \rceil$
        Get shift vector: $\Delta p = C[idx_x, idx_y, idx_z]$
        Calculate magnitude: $m = \|\Delta p\|^2$
        if $m < lower\_bound^2$ **then**
            $\Delta p \leftarrow 0$
        $local\_diff[i] = p_i - \Delta p$
        $local\_sq\_sum \mathrel{+}= \|\Delta p\|^2$
    Global reduce: $global\_diff \leftarrow$ MPI_Allreduce($local\_diff$)
    Global residual: $global\_sq\_sum \leftarrow$ MPI_Allreduce($local\_sq\_sum$)
    Update points: $Y \leftarrow Y - step\_size \cdot global\_diff$
    Check convergence: $\|global\_diff\| < convergence$
Master Process: Save final Y to point workspace

C. Parallel Strategy for Alignment

C.1. Performance Bottlenecks in Local Alignment

CryoAlign's local alignment method achieves precise matching by framing it as a global retrieval problem within a smaller "dataset." Initially, a translation mask segments the larger point cloud, followed by a two-stage alignment process to compute transformation matrices. Similarity scores are calculated for all overlays, and the best score is selected as the output. The local alignment involves iterative computations, where the translation mask traverses the volume along the x, y,

and z axes. As illustrated in Figure S1, this process has a time complexity of O(x_step * y_step * z_step), which can become a performance bottleneck when dealing with large density maps.

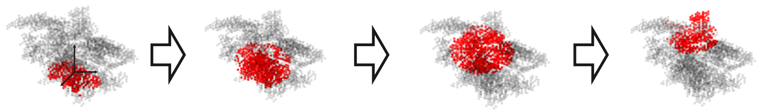

Fig. S1. The alignment process in local alignment.The gray point cloud represents the overall point set, while the red point cloud represents the local point set. Taking the z-axis as an example, the local point set within a certain range (spherical mask) is traversed along the z-axis of the overall point set at a specific step size. Each time the local point set moves, an alignment operation is performed.

To improve efficiency, we employs a hybrid parallel strategy using MPI and OpenMP. MPI provides coarse-grained parallelism for the point cloud registration process, further enhancing alignment efficiency. OpenMP, designed for shared memory parallel systems, parallelizes the triple loop of the translation mask by distributing iterations across threads, significantly reducing runtime. Algorithm S2 commences with the initialization of the MPI environment, where in computational processes are configured and distributed across available resources. Subsequently, the algorithm orchestrates the partitioning and distribution of input data, specifically including B_pcd, B_key_pcd, A_key_pcd, A_key_feats, and B_key_feats, to all participating processes through MPI communication mechanisms. Each process independently operates on its allocated subset of data by extracting point cloud structures and their respective keypoints, performing parameter extraction for the translation mask, verifying the dimensional integrity of the mask, and conducting local feature extraction to establish correspondences between keypoints in the input datasets. Once the correspondences are identified, the individual results from all processes are aggregated using MPI's collective communication protocols and transmitted to the root process.

At the root process, the algorithm constructs correspondence matrices based on these results, formulates the optimization problem required to compute an initial transformation matrix, and solves it to derive the preliminary transformation parameters. To enhance the alignment precision, the root process applies the Iterative Closest Point (ICP) algorithm, which iteratively minimizes the geometric distance between the transformed source point cloud and the target point cloud. Upon achieving convergence, an alignment score is calculated to quantitatively evaluate the quality of the transformation. Finally, the computed transformation matrix and the alignment score are broadcast to all processes using MPI's communication primitives to ensure global consistency. The algorithm concludes with the finalization of the MPI environment, ensuring that all resources are properly released and the distributed processes are cleanly terminated. This structured workflow enables efficient parallel computation and robust point cloud registration, particularly for large-scale datasets.

C.2. Parallel Registration Algorithm Description

The hybrid MPI-OpenMP registration algorithm operates through five interconnected phases, combining distributed computing with shared-memory parallelism.

Phase 1: MPI Initialization and Data Preparation begins with the master process (rank 0) initializing the MPI environment and loading source (A_pcd) and target (B_pcd) point clouds. SHOT feature descriptors are computed for both clouds using spherical histograms with radius *feature_radius* × VOXEL_SIZE, capturing local geometric patterns through radial bins. Spatial validation is performed by comparing normalized bounding box dimensions through the inequality

$$\left|\frac{1}{3}\sum(A_{\max}-A_{\min})-\frac{1}{3}\sum(B_{\max}-B_{\min})\right|>\epsilon \quad (\epsilon=10^{-6}) \tag{S1}$$

terminating execution with dimension mismatch warnings when violated.

Phase 2: Hierarchical Parameter Distribution employs a two-stage broadcast mechanism for the `BroadcastParams` structure. Scalar parameters including spatial boundaries (**center**, **terminal**), search radius r, and step size Δ are transmitted via standard `MPI_Bcast` operations. Feature matrices $\mathbf{F}_A$ and $\mathbf{F}_B$ undergo specialized distribution through a custom `broadcast_matrix` lambda function that first communicates matrix dimensions (rows/cols) before broadcasting

Algorithm S2. Parallel Registration with MPI and OpenMP

Input: Data directories, parameters (VOXEL_SIZE, feature_radius, etc.)
Master Process:
Initialize MPI environment
Get world_rank and world_size
Define `BroadcastParams` structure
if world_rank == 0 **then**
    Load sample data (A_pcd, B_pcd)
    Compute SHOT features (A_key_feats, B_key_feats)
    Calculate processing boundaries and validation
Broadcast `BroadcastParams to all processes:`
Divide i-axis workload among MPI ranks
Initialize local storage for results
Begin OpenMP parallel region:
`#pragma omp for collapse(3)` (i, j, k loops)
for each spatial coordinate (i, j, k) **do**
    Create spherical mask
    Perform `Registration_mask` with ICP
    Store transformation matrix and score
End OpenMP parallel region
if world_rank == 0 **then**
    Collect results from all MPI ranks
    Save aggregated results to files
else
    Send local results to rank 0
Finalize MPI environment

row-major formatted Eigen::MatrixXd data blocks, enabling dynamic buffer resizing on receiver nodes.

Phase 3: Workload Partitioning strategically divides the i-axis search space $[0, \mathbf{terminal}_x]$ across MPI ranks using chunked allocation. The chunk size per rank is calculated through

$$\text{chunk} = \left\lfloor \frac{\mathbf{terminal}_x}{\text{world\_size}} \right\rfloor + \delta(\text{rank} < \mathbf{terminal}_x \mod \text{world\_size}) \tag{S2}$$

where δ represents an indicator function assigning residual chunks to lower-ranked processes. Each rank subsequently computes local boundaries as $\text{start\_i} = \text{rank} \times \Delta + \xi$ and $\text{end\_i} = \min(\text{start\_i} + \text{chunk}, \mathbf{terminal}_x)$, where ξ denotes initial offset compensation.

Phase 4: Multi-Grained Parallel Execution leverages OpenMP's `collapse(3)` directive to parallelize the three-dimensional (i,j,k) loop nest. Each thread generates spherical masks centered at $\mathbf{c}_{\text{mask}} = \mathbf{center} + (i\Delta, j\Delta, k\Delta)$ with radius r, then executes constrained ICP registration through `Registration_mask`. This process incorporates correspondence filtering beyond `max_correspondence_dist`, SHOT-based feature matching for initial alignment, and voxel-adaptive threshold adjustments. Successful transformations $\mathbf{T}_l \in \mathbb{R}^{4\times4}$ and fitness scores s_l are cached in thread-local buffers using Eigen matrices, with critical section protection during global result aggregation.

Phase 5: Result Aggregation implements non-blocking MPI communication with four distinct message tags. Coordinate-score pairs (`MPI_INT[3]`, `MPI_DOUBLE`) stream through tags 1-2, while transformation matrices serialize as `MPI_DOUBLE[16]` arrays via tags 3-4, preserving column-major ordering through explicit transposition:

$$\mathbf{T}_{\text{store}} = \mathbf{T}^T \quad \forall \mathbf{T} \in \text{local\_T} \tag{S3}$$

The master process asynchronously receives and concatenates these data streams, writing final results to `record_cpp.txt` (tab-separated coordinates/scores) and `record_T_cpp.npy` (NPY-formatted transformation tensors), achieving $\mathcal{O}(N/(P \times T))$ complexity scaling where P denotes MPI ranks and T OpenMP threads per node.

2. EXPERIMENTS

A. Dataset of density maps

To assess the performance of our retrieval system, we collected low-temperature electron microscopy images from a dataset provided by VESPER, specifically curated for both global and local density map retrieval Joseph et al. (2017). Initially, we excluded images with a resolution below 10 Å, focusing instead on those with a resolution above 10 Åand ensuring that each class contained more than five density maps. This selective filtering resulted in two distinct datasets for evaluating retrieval performance: the global matching retrieval dataset, which includes 75 Cryo-EM density maps across 15 classes, and the local matching retrieval dataset, featuring 181 Cryo-EM density maps across 24 classes. In addition, we achieved cross mixing of tasks between global and local matching retrieval to comprehensively evaluate the effectiveness and robustness of retrieval applications. We used unlabeled categories for data augmentation, which means that the category of the density map is unknown. The table S1 presents the resolution distribution for both the global matching retrieval and the local matching retrieval datasets. "Res. range" refers to the resolution range, "Cross dataset" denotes the inclusion of data from different sets for testing, and "Unlabelled map" indicates density maps categorized without specific labels. The counts of density maps are based on the resolution ranges provided. The unlabeled dataset construction protocol utilizes Cryo-EM density maps of low (>10Å), medium (5-10Å), and high resolution (<5Å) derived from the labeled VESPER dataset, while incorporating data from both global and local retrieval tasks. Notably, the unknown-label retrieval experiment is designed not to identify map categories, but to simulate real-world scenarios where map class affiliations are undisclosed, thereby testing the application's similarity retrieval performance under practical conditions.

Table S1. Resolution distribution of labeled and unlabeled maps

	**Labeled Map**	
**Res. range**	**Global retrieval**	**Local retrieval**
<5 Å	30	80
5-10 Å	30	70
Cross dataset	15	31
	**Unlabeled Map**	
**Res. range**	**Global retrieval**	**Local retrieval**
<5 Å	66	78
5-10 Å	187	210
>10 Å	67	112

B. The effectiveness of the scoring function

Our experiments are structured around three key retrieval tasks: global retrieval, local retrieval, and similarity retrieval involving density maps of unknown categories. For the global retrieval task, we utilized several retrieval strategies including global matching, local matching, and a hybrid approach combining both global and local methods.

The scoring function of CryoAlign2 integrates four components: normal cosine similarity, density consistency, SHOT features, and geometric discrepancy. To systematically evaluate the contribution of each component to the overall retrieval F1-score, we conducted ablation experiments on 105 density maps organized into 21 categories (5 maps per category), comprising 65 global retrieval instances and 40 local retrieval instances. The ablation study results (Table S2) demonstrate critical dependencies between metric components and their collective impact on retrieval performance.

Our experiments systematically reveal the weight allocation rationale for the four features in CryoAlign2's scoring function through single-metric performance tests and component ablation experiments. The single-metric experiments show that **Normals** and **SHOT features** achieve F1-

scores of 88.25% and 89.87% respectively when used independently, significantly outperforming **density consistency** (82.57%) and **geometric discrepancy** (82.14%). This indicates the superior discriminative power of the former two features for structural matching, directly supporting the rationality of assigning higher weights (0.3 each) to Normals and SHOT in the baseline configuration. Meanwhile, density and geometric discrepancy require more cautious weight allocation due to their dependency on prior constraints from other features.

In component ablation experiments, the removal of Normals (-Normals) reduces the F1-score to 85.47%. Despite increasing SHOT weight to 0.4, density to 0.45, and geometric discrepancy to 0.15, the performance loss remains unrecovered, confirming Normals' irreplaceable role in global spatial correspondence and justifying its minimum weight threshold of 0.3 to maintain directional constraints. When SHOT features are removed (-SHOT), although boosting Normals weight to 0.5, density to 0.3, and geometric discrepancy to 0.2 maintains the F1-score at 89.72%, the 2.64 percentage point drop from baseline demonstrates that SHOT's local geometric discrimination capability, while partially compensable through enhanced global features, still requires weight 0.3 to handle complex topological structures. Density removal (-Density) causes F1-score to plummet to 83.11% even with SHOT weight increased to 0.45 and geometric discrepancy to 0.3, validating density's auxiliary value as a global statistical metric that necessitates weight preservation in the 0.25-0.3 range. Geometric discrepancy removal (-Geometric) leads to 6.75 percentage point F1-score degradation despite increasing SHOT to 0.45 and density to 0.35, corroborating its role as a regularization term that should be capped at 0.15 weight to avoid over-suppression of valid matches.

The uniform weight configuration (0.25*4) achieves only 87.23% F1-score, 5.13 percentage points lower than baseline, highlighting the necessity of asymmetric weighting strategies. The baseline configuration (0.3/0.3/0.3/0.1) optimizes performance through synergistic mechanisms: Normals and SHOT establish dual spatial alignment via global directional constraints (0.3 weight) and local geometric descriptors (0.3 weight) respectively, density provides global distribution verification (0.3 weight), while geometric discrepancy suppresses outlier matches (0.1 weight). This configuration balances core feature discriminability with dynamic weight equilibrium, achieving 92.36% F1-score while mitigating overfitting risks. Experimental data conclusively dictate future parameter optimization boundaries: Normals0.3, SHOT0.3, density0.25-0.3, and geometric discrepancy0.15, ensuring maximal multi-metric synergy.

Table S2. Ablation Study: Impact of Different Metric Combinations on Retrieval F1-Score

Group	Metric Composition	Weight Allocation				F1 (%)
		Normals	Density	SHOT	Geometric	
Baseline	Full Metrics	0.3	0.3	0.3	0.1	**92.36**
Single Metric	Normals Only	1.0	0.0	0.0	0.0	88.25
	Density Only	0.0	1.0	0.0	0.0	82.57
	SHOT Only	0.0	0.0	1.0	0.0	89.87
	Geometric Only	0.0	0.0	0.0	1.0	82.14
Single Removal	-Normals	0.0	0.45	0.4	0.15	85.47
	-Density	0.25	0.0	0.45	0.3	83.11
	-SHOT	0.5	0.3	0.0	0.2	89.72
	-Geometric	0.25	0.35	0.45	0.0	85.61
	Uniform Weights	0.25	0.25	0.25	0.25	87.23

Figure S2 demonstrates the score distributions of the multi-dimension scoring function for global and local retrieval tasks. For the global retrieval task, the dataset comprises 15 categories with 5 mutually similar maps per category. The local retrieval task dataset contains 24 classesclass 1 data originates from a cross-validation dataset, including Class1 with 39 mutually local or global similar maps. The experimental objective requires each density map to accurately identify

its corresponding category. An ideal scoring function should yield scores approaching 1 for intra-class density maps and scores approaching 0 for inter-class maps. Similarity retrieval was performed using weighted criteria: (Normals 0.3, Density 0.3, SHOT 0.3, Geometric 0.1).

Figure S2a demonstrates the score distribution of the global retrieval task using the multi-dimension scoring function, showing its capability to effectively distinguish intra-class density maps (scores approaching 1) from inter-class samples (scores approaching 0). By integrating multiple similarity dimensions of registered point clouds, this method eliminates potential bias and exhibits a more concentrated distribution, significantly enhancing discriminative power between homologous and heterologous classes. A similarity threshold of 0.45 is proposed: scores above 0.45 indicate similarity, while scores below 0.45 signify dissimilarity. Figure S2c displays the multi-dimension score heatmap, where diagonal regions (intra-class comparisons) are markedly higher than other areas, indicating that mutually similar maps achieve higher scores when compared within the same class. This confirms our retrieval application's capacity for precise similarity scoring. Figure S2b presents the score distribution for the local retrieval task. The multi-dimension scores show greater concentration, with a similarity threshold set at 0.4: scores above 0.4 indicate similarity, and scores below 0.4 indicate dissimilarity. Figure S2d illustrates the dataset-wide matching score heatmap, where diagonal values represent intra-class scores (approaching 1) and off-diagonal regions denote inter-class scores (approaching 0). Notably, Class 1 data originates from a large cross-validation dataset, and despite volumetric discrepancies, our tool maintains fundamental retrieval and classification performance.

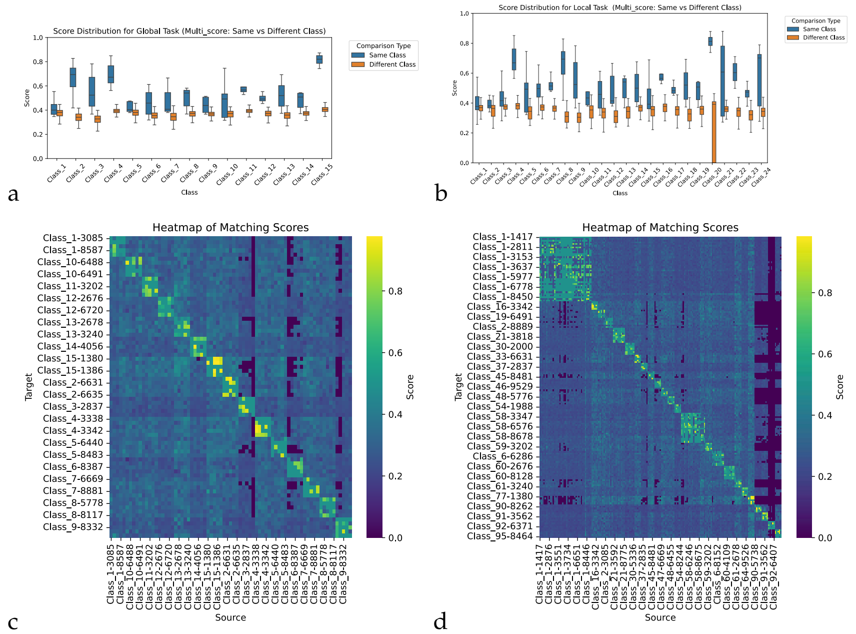

Fig. S2. The effectiveness of the similarity scoring function. **a** The score distribution of global retrieval task,based on multi-dimension evaluation scores. **b** The score distribution of local retrieval task, based on multi-dimension evaluation scores. Both **a** and **b**: Blue represents comparisons within the same category, Orange represents comparisons between different categories, **c** The heatmap of global retrieval task. **d** The heatmap of local retrieval task.Both **c** and **d**: The heatmap uses multi-dimension evaluation scores, with the diagonal representing scores within the same category, and the remaining areas representing scores between different categories.

Figure S3 showcases examples from our tool, demonstrating the effectiveness of our scoring function in both global and local retrieval tasks. In Figure S3a, for the global retrieval task, Ours, VESPER, Omokage, and EM-SURFER were used to search for EM maps similar to EMD-6631, returning the top 4 most similar density maps. It can be observed that Ours, VESPER, and Omokage successfully identified density maps similar to EMD-6631. Meanwhile, EM-SURFER made misclassifications, such as EMD-3341, which is not in the same category as EMD-6631 but received a high score. Since VESPER's scores are not initially normalized between 0 and 1,

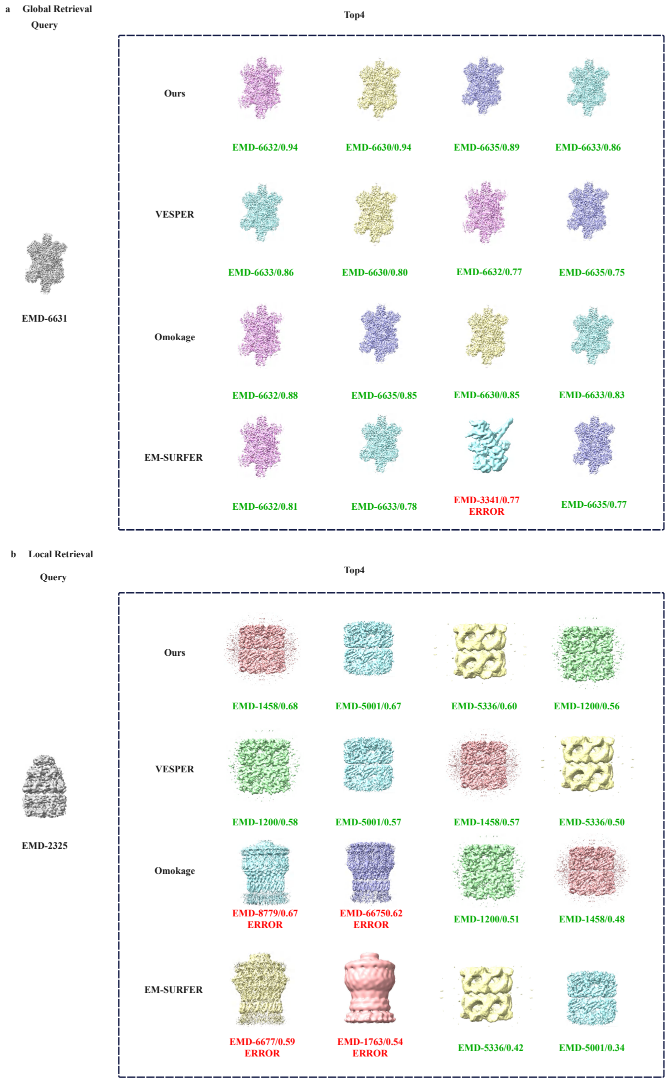

Fig. S3. The retrieval performances in different scenarios: **a** The global retrieval performance: the classification of EMD-6631 is dehydrogenase_GDH. Green indicates successful retrieval, while red indicates incorrect retrieval. After performing global retrieval using four applications, the top 4 results (EMD-6632/EMD-6630/EMD-6633/EMD-6635) retrieved by Ours/VESPER/Omokage all belong to the dehydrogenase_GDH category. However, EM-SURFER retrieved density maps from other categories with high scores instead. Although VESPER and Omokage also correctly identified the top 4 results, the normalized score distribution (score values following each EMD ID) obtained from our tooltool is higher than those from VESPER and Omokage. This indicates that our retrieval method has better discriminative capability. **b** The local retrieval performance: the classification of EMD-2325 is chaperone_GroEL. Green indicates successful retrieval, while red indicates incorrect retrieval. Similarly, after performing local retrieval using the four applications, the top 4 results (EMD-1458/EMD-5001/EMD-5336/EMD-1200) retrieved by Ours/VESPER all belong to the chaperone_GroEL category. However, Omokage/EM-SURFER made misclassifications, where density maps from other categories such as EMD-8779/EMD-1763 achieved high scores, while density maps from the same category such as EMD-1200/EMD-1458 did not score high. Furthermore, the normalized score distribution obtained from our tool is still higher than that of VESPER. Our scoring function demonstrates higher confidence in evaluating local similarities.

normalization is required. In the global retrieval task, the top 4 results from Ours have similarity scores closer to 1 compared to other applications and correctly identified the category, indicating the effectiveness and confidence of our tool in global retrieval tasks. However, the differences between our application, VESPER, and Omokage in global retrieval tasks are minimal. This also explains the reason why Omokage and EM-SURFER's F1 scores improve as the number of density maps in the global retrieval increases. At the same time, it also highlights that Omokage and EM-SURFER are only suitable for global retrieval tasks.

Figure S3b highlights the effectiveness of local retrieval. Similarly, using EMD-6631 as the query, although the top 4 results from Ours and VESPER all identified density maps of the same category as EMD-6631, the scores of Ours are closer to 1 compared to VESPER. This shows that our scoring function is more sensitive to the local similarity features of the density maps, demonstrating a higher confidence in local retrieval scores. For instance, with EMD-6631 as an example, the top 4 average score of VESPER is 0.55, while the average score of Ours is 0.62, indicating that our tool better evaluates the local similarity of density maps. In contrast, for Omokage and EM-SURFER, their retrieval results are still influenced by overall shape similarity, leading to misclassifications. For example, different-category density maps like EMD-6675 and EMD-1763 received high scores, while same-category density maps like EMD-5336 and EMD-1200 received low scores. Similarly, this phenomenon also explains why the F1 scores of Omokage and EM-SURFER decrease as more density maps are added to the local retrieval process. This is because their similarity scoring lacks sufficient discriminative power, resulting in misjudgments during local retrieval.

C. Impact of alignment methods and scoring Functions

Table S3. Decoupling Experiment Results: Alignment vs. Scoring Effects

**Group**	**Scoring Config**	**RMSD (Å)**	**F1-score (%)**
A1: Perfect	Default	0.5	88.57
A2: Perfect	Baseline	0.5	91.26
B1: Low Perturbation	Default	1.0	77.53
B2: Medium Perturbation	Default	5.0	61.49
B3: Medium Perturbation	Baseline	5.0	58.90
B4: High Perturbation	Default	10.0	34.68

1 **Group**: A-series (perfect alignment); B-series (perturbed alignment)
2 **Scoring Config**:
Default: Uniform weights (Normals 0.25, Density 0.25, SHOT 0.25, Geometric 0.25)
Baseline: Optimized weights (Normals 0.3, Density 0.3, SHOT 0.3, Geometric 0.1)
3 **RMSD**: Root Mean Square Deviation between aligned structures

We conducted controlled decoupling experiments to evaluate the independent effects of alignment quality and scoring functions on Cryo-EM density map retrieval performance. The study utilized 50 Cryo-EM map pairs processed through two protocols:**Dataset A (Perfect Alignment)**: Maintained ground-truth alignment matrices (RMSD = 0.5 Å) generated by CryoAlign2 while varying scoring function weights between default (Normals:0.25, Density:0.25, SHOT:0.25, Geometric:0.25) and baseline configurations (Normals:0.3, Density:0.3, SHOT:0.3, Geometric:0.1).**Dataset B (Perturbed Alignment)**: Applied Gaussian noise perturbations to ground-truth alignments at three RMSD levels (low: 1.0 Å, medium: 5.0 Å, high: 10.0 Å) while keeping scoring functions fixed.

The experiment simulated varying degrees of translational errors by modifying spatial position parameters in Cryo-EM alignment matrices. First, the three-dimensional translation vectors containing precise coordinates along the X, Y, and Z axes were extracted from original alignment data Punjani et al. (2017). Gaussian random noise was independently generated for each axis based on predefined perturbation intensity criteria. The amplitude distribution characteristics of the noise were governed by target root mean square deviation (RMSD) levels, with low-error conditions (1.0 Å) producing subtle positional fluctuations, medium levels (5.0 Å) introducing visible offsets, and high levels (10.0 Å) creating significant displacements. The generated noise

vectors were computationally superimposed onto original coordinates to create perturbed positional parameters. Rotation parameters remained strictly unchanged throughout this process to preserve structural orientation integrity. Each original alignment matrix was perturbed multiple times per error level, ensuring reproducibility through fixed random seeds. Post-perturbation validation automatically quantified actual translational deviations. Spatial distance comparisons between original and perturbed coordinates confirmed whether injected noise met predefined accuracy thresholds.

As shown in Table S3, alignment quality demonstrated significantly greater impact on retrieval performance than scoring function optimization:The F1-score decreased dramatically from 88.57% to 77.53% (12.5% relative reduction) when alignment error increased from 0.5 Å to 1.0 Å. Medium perturbations (5.0 Å) caused further degradation to 61.49% (30.7% total reduction), while severe misalignment (10.0 Å) reduced performance to 34.68%. Scoring function optimization provided limited benefits, improving F1-score by only 2.69% (88.57% to 91.26%) under perfect alignment. This gain became insignificant compared to alignment-induced losses - the 12.5% performance drop from 1.0 Å perturbation exceeded the maximum scoring function benefit by 4.6. At 5.0 Å RMSD, optimized scoring functions (B3 group) showed no statistically significant improvement over default parameters (B2 group).Statistical analysis revealed strong nonlinear correlation between F1-score and alignment accuracy, compared to weak linear correlation with scoring function weights. These results establish alignment quality as the dominant performance factor, particularly when RMSD exceeds 3.0 Å, beyond which scoring adjustments cannot compensate for alignment errors.

D. Unknown categories density maps retrieval

Figure S4 illustrates the similarity retrieval results of density maps from unknown categories. To validate the effectiveness of our tool, we added a large number of density maps from unknown categories into the database for hybrid retrieval tasks. By comparing Ours/VESPER/Omokage, we use EMD-6675 as an example to present the top 10 density maps with the highest similarity scores. Despite the presence of many low-resolution and small-volume density maps in the retrieval database, such as EMD-5722, EMD-8399, EMD-2629, EMD-3089, EMD-3087, and EMD-3088 shown in Figure S4a, our application is still able to capture local information and identify that these small-volume density maps share local similarity with the upper half of EMD-6675. This is further demonstrated in the third example "Ours Alignment", where it is evident that the upper half of EMD-6675 indeed shares local similarity with EMD-8399, which is ranked highly in the retrieval results. For density maps of the same category as EMD-6675, such as EMD-6676, EMD-8779, EMD-1763, and EMD-6677, these maps also achieve high scores. Additionally, our tool does not exhibit misjudgments when evaluating high-resolution, feature-rich density maps. There were no cases where high-resolution density maps with completely dissimilar shapes received abnormally high scores. This further confirms that our tool is more suitable for retrieving medium and high-resolution density maps. Compared to the retrieval results of VESPER and Omokage, as shown in Figure S4b, the accuracy of VESPER is lower than that of our tool, as it exhibits misjudgments and fails to effectively capture local features. Omokage, on the other hand, relies on shape and size similarity for retrieval. As illustrated in Figure S4c, the retrieval results of Omokage for EMD-1458 and EMD-5001 do not show obvious local similarity with EMD-6675. Their high retrieval scores are based solely on surface shape similarity with EMD-6675. This further indicates that Omokage is only suitable for global retrieval tasks and not for local retrieval tasks. In contrast, our tool is well-suited for hybrid retrieval tasks.

E. Correct alignment promotes accurate search

Figure S5 displays the maps visualized in superimposed states using ChimeraX. The fitmap uses local optimization methods to adjust the position and orientation of the model. If the initial pose is too far from the actual position of the target density map, the algorithm may get stuck in a local optimum and fail to find the global optimum. As shown in Figure S5(fitmap), if the front and back were reversed, the results can easily resemble those of EMD-8097/EMD-3356, resulting in poor overlay outcomes. Since our method uses a point-set matching method for comparison, our tool does not require an initial pose and it can generate a RT matrix, which allows us to transform the position of the source map to achieve its transformed pose. The first column of Figure S5 shows the optimal transformation matrix obtained using our tool, followed by the rotation and translation applied to the source map. The second column displays the optimal transformation matrix obtained using VESPER, along with the resulting overlay after applying the rotation and

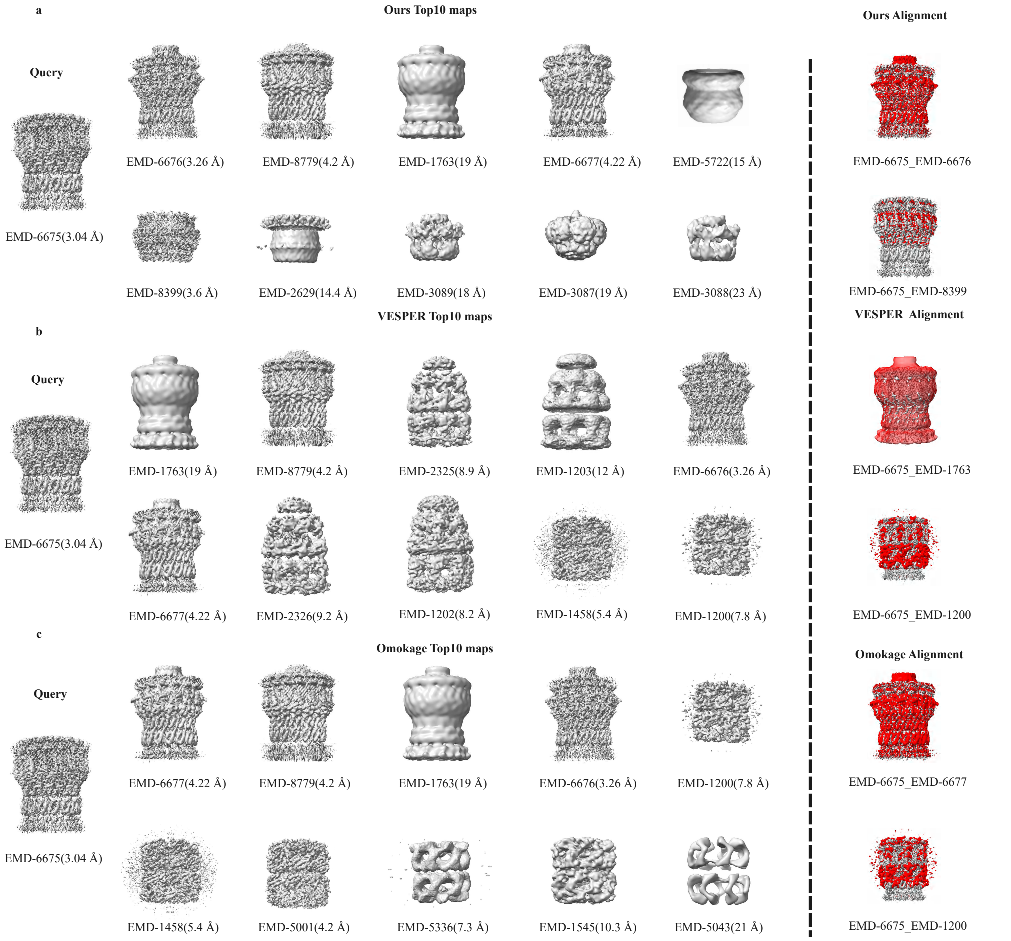

Fig. S4. The results of hybrid retrieval task. Using EMD-6675 as an example, perform a hybrid retrieval using different retrieval applications. The results are ranked based on similarity scores, with density plots of higher similarity positioned further to the left, and the first row having higher similarity than the second row. The third column shows the alignment of density maps using the RT matrices obtained from the results of Ours/VESPER/Omokage retrievals. The red color represents the query map, and the gray color represents the retrieved result map. **a** The top 10 density plots from the hybrid retrieval results of EMD-6675 using our tool. As shown by the alignment, for global retrieval tasks with small volume differences, Ours achieves good alignment. For local retrieval tasks with significant volume differences, such as EMD-6675_EMD-8399, it also achieves satisfactory alignment. **b** The top 10 density plots from the hybrid retrieval results of EMD-6675 using VESPER. For global retrieval tasks with small volume differences, VESPER achieves good alignment. However, VESPER fails to retrieve the locally similar EMD-8399, and as shown by the alignment, EMD-1200 does not exhibit high similarity to EMD-6675. **c** The top 10 density plots from the hybrid retrieval results of EMD-6675 using Omokage. For global retrieval tasks with small volume differences, Omokage achieves good alignment. Similarly, Omokage assigns a high score to EMD-1200, but after alignment, it is evident that EMD-1200 does not exhibit high similarity to EMD-6675.

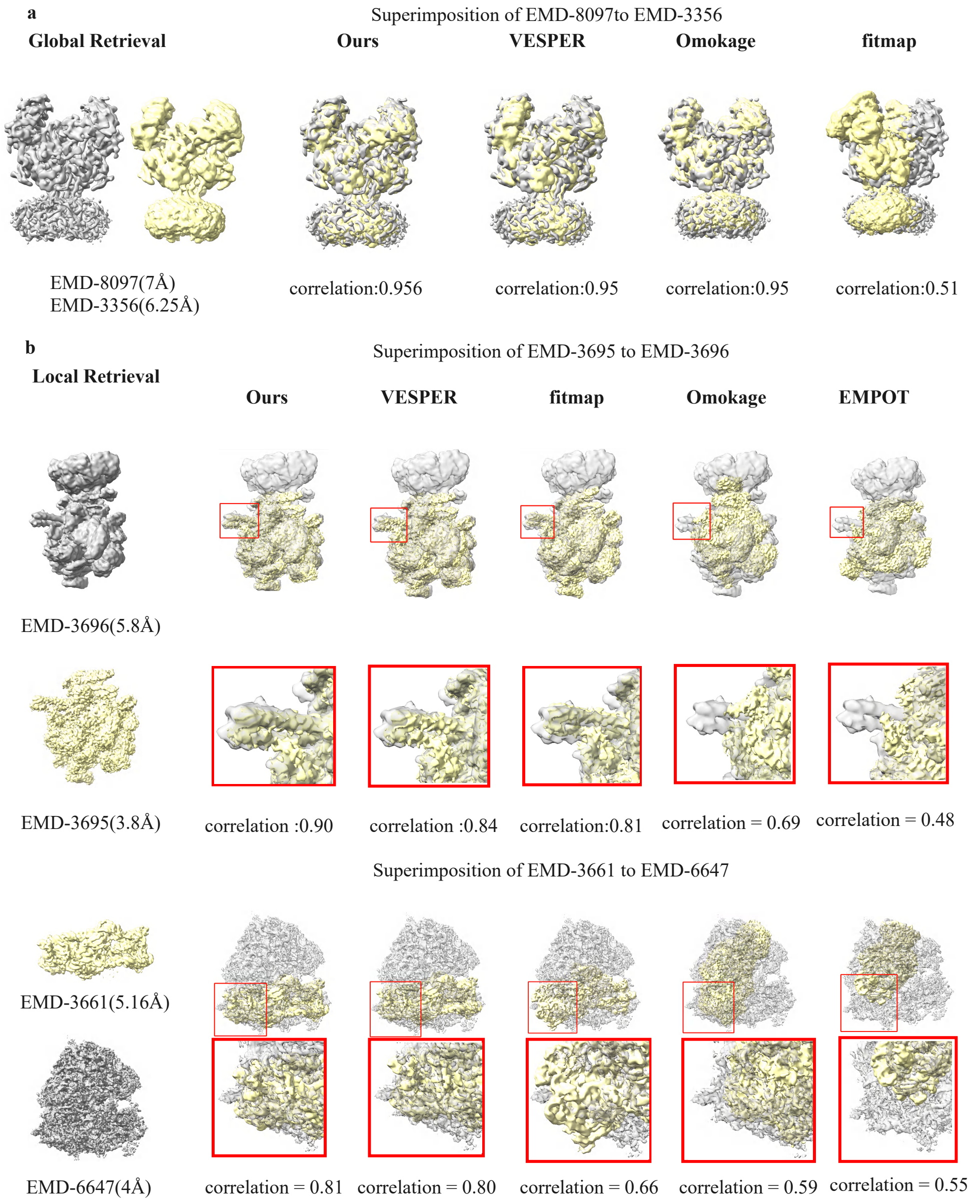

Fig. S5. The superimposing results using estimated alignment parameters. **a** An example of overlaying global retrieval result density plots, using EMD-8097 and EMD-3356 as examples. The correlation of Ours/VESPER/Omokage/fitmap is 0.956/0.95/0.95/0.51. **b** An example of overlaying local retrieval result density plots, using EMD-3696 and EMD-3695 as examples. The correlation of Ours/VESPER/fitmap/Omokage/EMPOT is 0.90/0.84/0.81/0.69/0.48. Using EMD-3661 and EMD-6647 as examples, the correlation of Ours/VESPER/fitmap/Omokage/EMPOT is 0.81/0.80/0.66/0.59/0.55.

translation. It is clear that our tool yields the best accuracy in the overlay process.

For the global retrieval, since involved maps have similar sizes, the overlay effects using the RT matrices generated by VESPER, Omokage, and our tool for EMD-8097/EMD-3356 show minimal differences. However, for local retrieval tasks, it is evident that Omokage is not suitable. Omokage still relies on global shape characteristics and fails to capture local feature information, resulting in poor performance when faced with significant volume differences. Taking EMD-3695/EMD-3696 as an example, the overlay effect using our provided RT matrix is slightly better than VESPER's. VESPER's result tends to deviate outward, with a correlation of 0.84 after applying fitmap, while our tool achieves a correlation of 0.90 after the overlay. In the case of EMD-3661/EMD-6647, the overlay effect using our provided RT matrix also slightly outperforms VESPER, which shows an upward deviation and achieves a correlation of 0.80, while our tool yields a correlation of 0.81. Compared with EMPOT Riahi et al. (2023), a local registration tool that also employs point cloud sampling techniques, our method demonstrates enhanced robustness in cross-coordinate-system scenarios. When processing unaligned density maps (e.g., EMD-3695/3696 and EMD-3661/6647 cases with initial poses distributed across distinct coordinate systems and exhibiting significant rotational/translational differences), EMPOT's sampled point clouds are vulnerable to peripheral noise interference. Although the algorithm includes normalizing both point clouds to the origin coordinate system, spatial disturbances from noisy environments lead to substantially increased failure rates during keypoint correlation matching, resulting in deteriorated registration accuracy. Our method addresses this limitation by integrating the SHOT feature descriptor, which effectively captures local geometric characteristics through 3D orientation histogram-based feature representation, enabling stable feature matching in noisy conditions.

F. Robustness of our retrieval tool

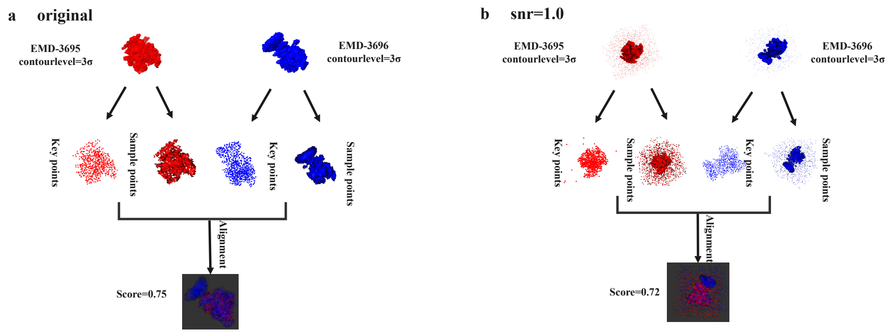

Fig. S6. The example (EMD-3695, EMD-3696) for low SNR **a** At SNR=1, point cloud sampling and critical point extraction were performed on EMD-3695 and EMD-3695 with contour level set to 3σ, yielding an alignment score of 0.72. **b** At origin map, point cloud sampling and critical point extraction were performed on EMD-3695 and EMD-3695 with contour level maintained at 3σ, resulting in an alignment score of 0.75.

Table S4. Alignment Performance Metrics Across SNR Levels

SNR	Score	Angular Discrepancy (°)	Translational Discrepancy (Å)
5.0	0.75	0.51	0.78
2.0	0.73	1.35	1.27
1.0	0.72	1.87	1.69
0.5	0.42	3.14	10.56

To evaluate the robustness of our retrieval application, we conducted alignment experiments on EMD-3696 and EMD-3695 density maps under various SNR levels. As shown in Figure S6a (SNR=1.0 simulation), the method demonstrates significant noise tolerance: strategic sampling effectively isolates key point clouds while preserving molecular contour integrity, a prerequisite

for subsequent point cloud alignment. CryoAlign2 alignment achieves an evaluation score of 0.72, confirming spatial correspondence maintenance under strong noise. Figure S6b ("original map") shows the alignment score of 0.75 for pristine EMD-3695 and EMD-3696. Notably, the alignment score at SNR=1.0 (0.72) exhibits only a 0.03 decrease compared to the original benchmark, demonstrating our retrieval's applicability under SNR=1.0 conditions.

To systematically validate robustness, experiments were conducted at SNR levels of 0.5, 1.0, 2.0, and 5.0 using noise-added versions of EMD-3695 and EMD-3696. The rotation-translation (RT) matrix parameters from noisy alignments were compared with those from pristine alignments through angular and translational deviation calculations. As detailed in Table S4, our method maintains stable performance at high SNR. Quantitative analysis reveals minor RT matrix deviations between noise-perturbed and pristine benchmarks. Specifically, angular discrepancies remain below 1.87° and translational errors peak at 1.27 Å for SNR$\geq$1.0. At SNR=5.0, both angular (0.51°) and translational (0.78 Å) errors reach sub-angstrom precision.

The alignment score decreases progressively from 0.75 (SNR=5.0) to 0.42 (SNR=0.5). Remarkably, at SNR=1.0, the score remains high (0.72) with only 0.03 absolute decrease from the pristine benchmark, highlighting effective molecular contour identification. These findings collectively validate our method's robustness in retrieving high-resolution cryo-EM density maps, demonstrating effective scoring capability even at SNR=1.0. The approach maintains precision through optimized point cloud filtering under high SNR while achieving noise-agnostic alignment stability via shape descriptor invariance. This robustness proves particularly advantageous for processing experimental cryo-EM datasets containing unresolved regions and partial volume effects.

G. Running time analysis

The performance test primarily evaluates the comparison speed of the CryoAlign retrieval application. The experimental environment is configured with an Intel(R) Core(TM)i9-10900X CPU@3.70GHz, 64G RAM, and an NVIDIA GeForce RTX 3080 10G. The local alignment of CryoAlign is a refined approach that, when combined with the global alignment method, yields more accurate alignment results. More precise alignment is beneficial for the retrieval application of maps.

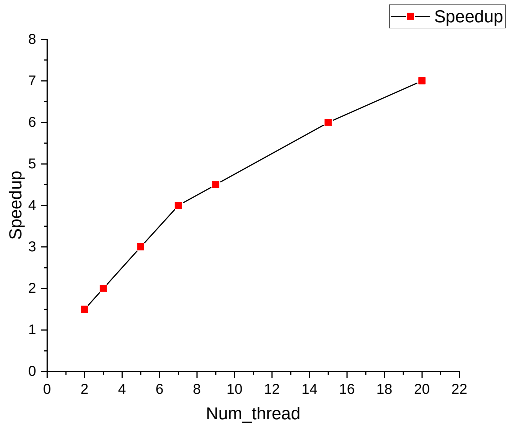

Fig. S7. The acceleration achieved through parallel processing

Taking EMD-3661 and EMD-6647 as examples, EMD-3661 samples 4,647 points while EMD-6647 samples 11,384 points. Local alignment with single-threaded processing creates a substantial performance bottleneck. As illustrated in Figure S8, parallel processing achieves significant acceleration: when utilizing 20 threads, the acceleration ratio reaches 1:7 compared to single-threaded alignment. This demonstrates that after parallel optimization, the execution efficiency of a single local alignment operation can reach 7 times that of pre-optimized processing.

When comparing performance using the same environment and data, CryoAlign2s accelerated local alignment method can reach speeds that are twice as fast as VESPER's, given the same number of threads. Table S5 presents the performance of the algorithm's parallel processing. We conducted performance tests before and after parallelization on three tasks: automated construction of point cloud libraries (75 Classes), keypoint extraction algorithm, and mask alignment algorithm. The performance optimization for automated construction of point cloud libraries

primarily involves parallel processing of large-scale data, with a speed increase of nearly 50% after parallelization. The keypoint extraction algorithm focused mainly on optimizing data transfer between the CPU and GPU, with significant optimizations made to the Meanshift algorithm, achieving a speed increase of nearly 45%. The mask alignment algorithm can be regarded as a set of multiple global alignment, and it is natural to use multi-core parallel acceleration.

Table S5. The performance comparison before and after parallelism

Task	Before Parallelism	After Parallelism
Create point DB		
(75 times)	36min	15min
Extract points	90s	50s
Mask alignment	1min30s	11s

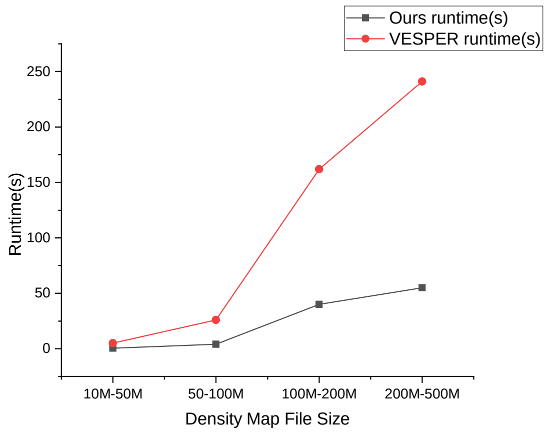

Fig. S8. The runtime comparison between our tool and VESPER. The black line represents the runtime of our tool, while the red line represents VESPER's runtime. Our application operates significantly faster when dealing with comparisons of large-volume density maps, achieving speeds up to four times faster than VESPER.

After comparing the runtime of our optimized CryoAlign with VESPER, we found that VESPER's alignment process tends to be significantly slower when handling larger volume density maps. Subsequently, we categorized the density maps by file size and selected 5 pairs of maps from the following ranges: 10M-50M, 50M-100M, 100M-200M, and 200M-500M. We ensured that both alignment applications were configured to use four threads and compared the runtime of CryoAlign and VESPER. The results, as shown in Figure S8, indicate that for larger volume density maps in the 200M to 500M range, which contain more complex feature information, the runtime of VESPER increases significantly, whereas our tool is much faster than VESPER. This also demonstrates that while VESPER is suitable for alignment tasks, its inefficiency in handling larger volume density maps makes it unsuitable for retrieval tasks.

REFERENCES

Chen, T.-Y., C.-C. Hsiao, and C.-C. Huang (2023). Density-imbalance-eased lidar point cloud upsampling via feature consistency learning. *IEEE Transactions on Intelligent Vehicles 8*(4), 2875–2887.

Joseph, A. P., I. Lagerstedt, A. Patwardhan, M. Topf, and M. Winn (2017). Improved metrics for comparing structures of macromolecular assemblies determined by 3d electron-microscopy. *Journal of Structural Biology 199*(1), 12–26.

Punjani, A., J. L. Rubinstein, D. J. Fleet, and M. A. Brubaker (2017). cryoSPARC: Algorithms for rapid unsupervised cryo-EM structure determination. *Nature Methods 14*(3), 290–296.

Riahi, A. T., C. Zhang, J. Chen, A. Condon, and K. D. Duc (2023). EMPOT: Partial alignment of density maps and rigid body fitting using unbalanced gromov-wasserstein divergence. *arXiv preprint arXiv:2311.00850*.

Salti, S., F. Tombari, and L. di Stefano (2014). Shot: Unique signatures of histograms for surface and texture description. *Comput. Vis. Image Underst. 125*, 251–264.

Wang, W., X. Lu, D. Shao, X. Liu, R. Dazeley, A. Robles-Kelly, and W. Pan (2023). Weighted point cloud normal estimation. In *2023 IEEE International Conference on Multimedia and Expo (ICME)*, pp. 2015–2020.
